# Efficient production of icariin and baohuoside I from *Epimedium Folium* flavonoids by fungal *α-*l*-*rhamnosidase hydrolysing regioselectively the terminal rhamnose of epimedin C

**DOI:** 10.1186/s13068-023-02348-6

**Published:** 2023-06-30

**Authors:** Shanshan Zhang, Changning Lu, Shiping Cao, Qi Li, Guangwei Wu, Linguo Zhao

**Affiliations:** 1 Jiangsu Co-Innovation Center of Efficient Processing and Utilization of Forest Resources, Nanjing Forestry University, Nanjing, 210037 China; 2 College of Chemical Engineering, Nanjing Forestry University, 159 Long Pan Road, Nanjing, 210037 China

**Keywords:** *α-*l-Rhamnosidase, Icariin, Baohuoside I, *Epimedium Folium* flavonoids, Biotransformation

## Abstract

Industrial application of icariin and baohuoside I has been hindered by the short supply to a great extent. In this work, a novel GH78 *α-*l-rhamnosidase AmRha catalyzed the bioconversion of low-value epimedin C in crude *Epimedium Folium* flavonoids (EFs) to icariin and baohuoside I was developed. Firstly, the high-level expression of *AmRha* in *Komagataella phaffii* GS115 attained an enzyme activity of 571.04 U/mL. The purified recombinant AmRha could hydrolyze *α-*1,2-rhamnoside bond between two rhamnoses (*α-*Rha(2 → 1)*α-*Rha) in epimedin C to produce icariin with a molar conversion rate of 92.3%, in vitro. Furtherly, the biotransformation of epimedin C to icariin by the recombinant *Komagataella phaffii* GS115 cells was also investigated, which elevated the EFs concentration by fivefold. In addition, biotransformation of epimedins A-C and icariin in the raw EFs to baohuoside I was fulfilled by a collaboration of AmRha and *β*-glucosidase/*β*-xylosidase Dth3. The results obtained here provide a new insight into the preparation of high-value products icariin and baohuoside I from cheap raw EFs.

**Supplementary Information:**

The online version contains supplementary material available at 10.1186/s13068-023-02348-6.

## Introduction

**Fig. 1:**
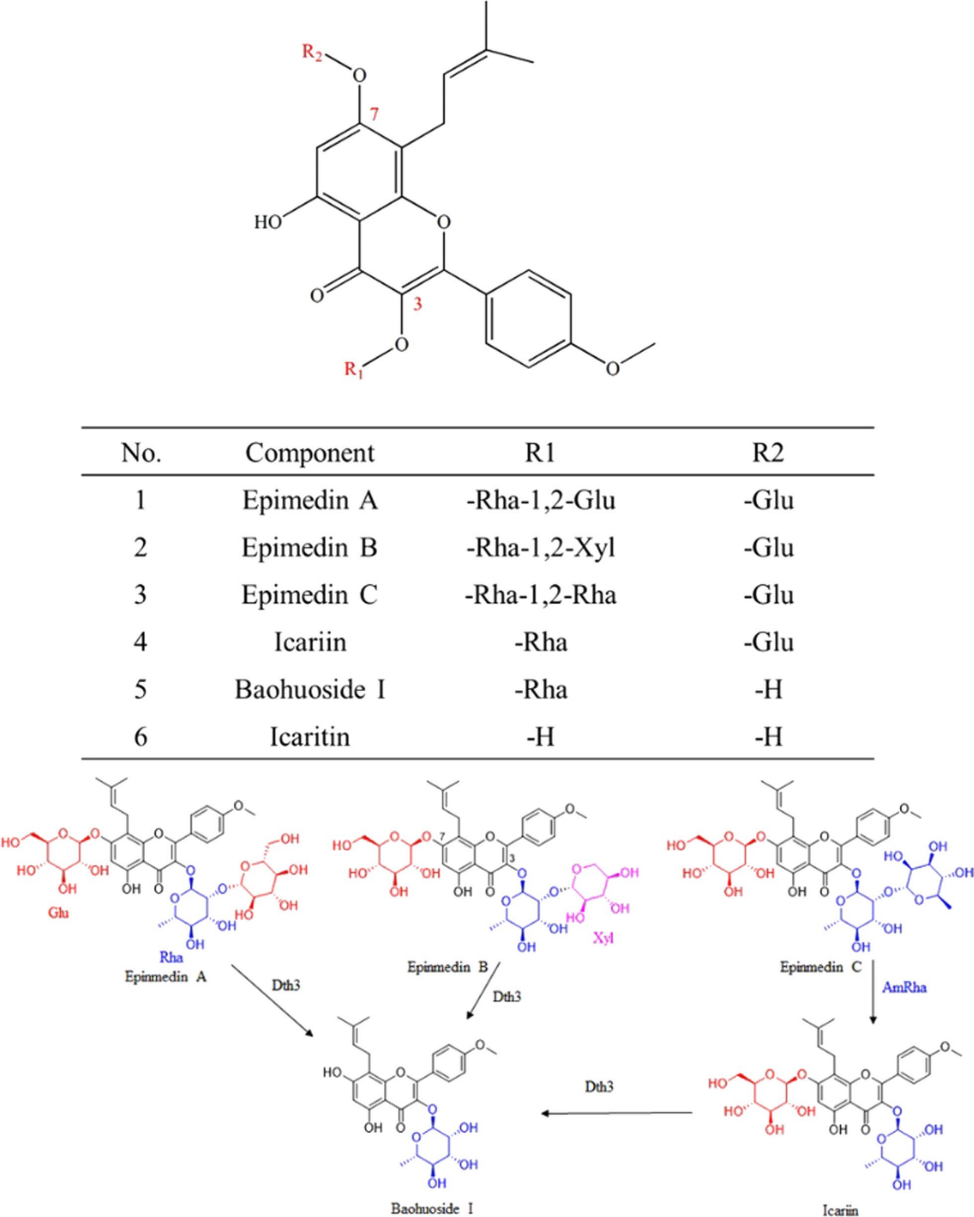
Structure of *Epimedium Folium* flavonoids and schematic diagram of transformation of EFs into baohuoside I

*Epimedium Folium* flavonoids (EFs) were reported as the significant pharmacological activity in *Epimedium Folium* which was used for a long time in TCM clinic [1, 2]. Generally, in the EFs, flavonoid glycosides with prenylation, including icariin, epimedin A, epimedin B, epimedin C and baohuoside I, are considered as the representative components [3–5]. Their structures are very similar. They share the same aglycone skeleton with the major difference in the sugar group linked at the C-3 or C-7 position. For instance, epimedin C possesses two rhamnose linked by *α-*1, 2-glycosidic bond at C-3 position, but icariin has only one rhamnose group at C-3 position (Fig. 1). EFs have been proved to exhibit broad biological activities. Particularly, icariin and baohuoside I are the most effective components, which have aroused great interest in many fields. Meanwhile, icariin has been written as the quality marker (Q-marker) of EFs in *Chinese Pharmacopoeia* [1]. Significant pharmacological activities of icariin and baohuoside I revealed the promising potential of drug candidates and tonics. For instance, icariin shows a significant curative effect on many diseases such as sexual dysfunction, osteoporosis, cancers, etc. [6–9]. Baohuoside I exhibited similar pharmacological activities with those of icariin, it is worth noting that baohuoside I shows the most significant effect on the treatment of osteoporosis in contrast to other bioactive constituents in EFs [10].

However, further industrial development of icariin and baohuoside I has been hindered by the lower yields to a great extent. It is noted that icariin contents in plants are extremely rare (only about 1%), which leads to the price of pure icariin is as high as $6000/kg [4]. Although the relative content of icariin in the crude extract of Epimedium is the highest, in comparison with other analogs in EFs, it is far from enough to meet the ever-increasing demand. Thus, it is urgently needed to enlarge producing sources of icariin. In industry, both icariin and baohuoside I are prepared from *Epimedium Folium*, which are usually extracted with an organic solvent to obtain crude EFs. The crude EFs suffered from multi-step purification processes to yield the targeted materials with low efficiency [11]. During the purification of them, the other components such as epimedins A–C in the crude EFs are generally discarded as waste. However, the content of epimedin C is usually the second-highest content, next only to that of icariin in the EFs, and even the highest content in *Epimedium* species in Shaanxi (China). Given the aforementioned facts, epimedin C could be an ideal substrate to transform to icariin and baohuoside I by hydrolyzing the terminal rhamnose group of epimedin C at the C-3 position.

Considering the low regioselectivity and possible environmental pollution of conventional acid-mediated hydrolysis of flavonoid glycosides, enzyme catalysis provides an eco-friendly alternative for bioconversion of epimedin C to icariin and baohuoside I [12, 13]. Previous studies suggested that releasing the terminal rhamnoside at C-3 in epimedin C is the rate-limiting step in bioconversion [14]. Mining the enzyme regioselectively hydrolysing *α-*1,2*-*rhamnosidic bond would be crucial to achieve the bioconversion. *α-*l*-*Rhamnosidase is an important enzyme that is responsible for releasing the terminal *α-*l*-*rhamnose from a series of glycosides, glycolipids and other natural products by hydrolysing various *α-*1*, α-*1,2*, α-*1,3*, α-*1,4 and *α*-1,6 glycosidic bonds [15, 16]. Therefore, screening of α-l-rhamnosidase that only hydrolyzing α-1,2-rhamnosidic bond between rhamnoside and rhamnoside is important to fulfil the bioconversion of epimedin C to icariin and baohuoside I. To date, only two such *α-*l*-*rhamnosidases were reported. However, owing to low enzyme activity and other factors (such as the transformation of suitable temperature and resistance to organic reagents), their industrial applications were limited [14, 17]. Thus, new *α-*l*-*rhamnosidase with strict specificity to *α-*1,2*-*rhamnosidic bond is worthy of developing to overcome the deficiencies of present enzymes.

Herein, a novel *α-*l*-*rhamnosidase AmRha from *Aspergillus mulundensis* was discovered through the bioinformatics analysis, protein expression and functional characterization. AmRha belongs to the GH78 family and could release specifically the terminal rhamnoside in epimedin C, leading to high-efficient production of icariin. To the best of our knowledge, the recombinant *α-*l*-*rhamnosidase AmRha presented the highest catalytic efficiency to transform epimedin C in raw EFs to icariin so far. The optimized reaction condition allowed us to achieve the efficient bioconversion of epimedin C to icariin in vitro. While, at the aid of a collaboration of AmRha and glucosidase Dth3 with *β*-xylosidase activity, subsequent biotransformation of epimedins A–C and icariin in the raw EFs to baohuoside I was also investigated in vitro. A strategy to remove the feedback inhibition of rhamnose was further established, through the whole-cell transformation of epimedin C to icariin in the recombinant engineered *Komagataella phaffii* AmRha-GS115 that could be tolerant to a high concentration of raw EFs.

## Experimental parts

### Materials

*p*-Nitrophenyl(*p*NP)*-α-*l*-*rhamnopyranoside (*p*NPR) (Lot No. N168186), *p*NP-*α-*l*-*arabinofuranoside (*p*NPAraf) (Lot No. M331421) and *p*NP*-α-*l*-*arabinopyranoside (*p*NPArap) (Lot No. N166589) were products from Shanghai Aladdin Biochemical Technology Co., Ltd. (Shanghai, China). *p*NP*-β-*d*-*glucopyranoside (*p*NPG) (Lot No. 141766941809154) and *p*NP*-β-*d*-*xylopyranoside (*p*NPX) (Lot No. C12089313), were purchased from Sigma-Aldrich Trading Co, Ltd (US). Epimedin A (Lot No. A0228, > 98%), epimedin B (Lot No. A0229, > 98%), epimedin C (Lot No. A0230, > 98%), icariin (Lot No. A0145, > 98%) and baohuoside I (Lot No. A0637, > 98%) (Spectrum pure, purity > 98%) were purchased from Chengdu Must Bio-Technology (Chengdu, China). The flavonoids mixture extracted from *Epimedium Folium* (purity of 12.76%, the main ingredients are epimedin A, epimedin B, epimedin C, epimedin and baohuoside I, production lot number: No. 20211211, No. 22-12-23) was donated by Jiangsu Kanion Pharmaceutical Co. Ltd. (Lianyungang, China) and Shaanxi Tianneng Co., Ltd. (Shaanxi, China).

*Escherichia coli* strain was incubated in the Luria–Bertani (LB) medium under the optimal conditions (0.01 mM isopropyl*-β-*d*-*thiogalactopyranoside was used as an inducer and induced at 28 °C for 14 h). The *E. coli* DH5α strain from Vazyme Biotech Co. Ltd. (Nanjing, China) was used as the cloning host. The expression host *Komagataella phaffii* GS115 was obtained from Microbiology Laboratory (Nanjing Forestry University, China).

### Plasmid constructions

The gene of *AmRha* (Genbank: XM_026751123.1) from *Aspergillus mulundensis* was synthesized by Generay Biotech Co., Ltd. (Shanghai, China) and inserted into the expression plasmid *pET-28a*(+). DNA manipulation was conducted by refs [18, 19]. The encoding gene *AmRha* was amplified by Prime STAR HS DNA polymerase (TaKaRa Bio, USA) with recombinant plasmid *pET-28a*(+)*-AmRha* as the template. The target gene was amplified by PCR with specific primers *AR-pPICZαA-f* and *AR-pPICZαA-r*. Primers *AR-pPIC-f* (CATGCCATGGCAATGAAGTCAAGTAATATTTACTC) and *AR-pPIC-r* (CCGCTCGAGTATCTTTTCCATAT) were used in PCR, which the restriction digestion sites were NcoI and XhoI. The PCR product was purified by a MinElute Gel Extraction Kit (Axygen, USA). Then, the purified target fragment was digested with DNA restriction enzymes NcoI and XhoI, and inserted into the digested vector *pPICZα*A (Novagen, Germany) by T4 DLigase (TaKaRa, China). The recombinant plasmid *pPICZα*A*-AmRha* was transformed into *E. coli* DH5α by heat shocking. The recombinant plasmid *AmRha-pPICZα*A was extracted by a Plasmid Miniprep Kit (Axygen, USA) and linearized with restriction enzyme MssI. Finally, the linearized recombinant plasmid *AmRha-pPICZα*A was transformed into *Komagataella phaffii* GS115 by electroporation.

### Sequence alignment, homology modeling and molecular docking

Databases: BLAST (www.blast.ncbi.nlm.nih.gov/Blast.cgi), CAZy (www.cazy.org) [20] and PDB (www.rcsb.org). Structural prediction softwares were PyMOL (www.pymol.org), Swiss Model (www.swissmodel.expasy.org) and I-TASSER (https://zhanglab.ccmb.med.umich.edu/I-TASSER/).

The molecular biology software DNAMAN 6.0 was used for sequence alignment blast and amino acid sequence analysis. Prediction of AmRha protein structure and the specific information of ligand binding site were carried out based on the I-TASSER server [21]. The 3D protein structure model of AmRha was constructed using the homology modeling by the Swiss Model. The predicted catalytic active center of AmRha was visualized by Swiss-PDB Viewer and PyMOL. The epimedin C structure was downloaded from PubChem and stored in SDF format [22]. The structures of *α-*l*-*rhamnosidase AmRha and epimedin C were pretreated by Schrodinger 2015, and the lattice file was prepared at the ligand binding sites of protein using Glide 6.6 [23]. Finally, the preprocessed small molecule epimedin C was spliced in the lattice file, and the predicted docking conformation was obtained.

### Expression and purification of AmRha

The recombinant *Komagataella phaffii* GS115 was cryopreserved by a cultivation as reported [20]. YPD and BMGY were used for the cultivation of *Komagataella* [20]. *Komagataella phaffii* GS115 was cultivated at 28 °C and 180 r/min [20].

The supernatant was collected by centrifugation (4 °C, 8000×*g*, 30 min) from the culture and precipitated with 80% ammonium sulphate. The enzyme precipitate was dissolved and dialyzed under the optimal conditions followed by ref [20]. The dialysate was loaded onto a column of DEAE Sepharose Fast Flow equilibrated with Tris–HCl buffer (50 mM, pH 7.5). The enzyme was eluted from the column using 20–300 mM NaCl and dialyzed by distilled water and phosphate buffer (10 mM, pH 6.5) at 4 °C for 4 h and 20 h, respectively. Finally, the purified enzyme was stored with 30% glycerinum at − 20 °C.

The protein analysis via SDS-PAGE was conducted. The protein concentration was determined by the Bradford protein assay kit (Sangon Biotech, China).

### Enzyme activity assay

The *α-*l*-*rhamnosidase AmRha and *β*-glucosidase Dth3 activity assay used *p*NPR and *p*NPG as the substrate, respectively. The reaction system was as follows: 1 mM substrate, 50 mM sodium phosphate buffer (pH 5.5 for AmRha and pH 5.0 for Dth3 [24]), and purified enzyme AmRha or Dth3. The reaction system of AmRha or Dth3 was incubated at 65 °C or 85 °C for 5 min, respectively [24]. Finally, the reaction was terminated by the addition of an appropriate amount of 1 M Na_2_CO_3_. The released *p*NP was assayed at 405 nm immediately. One unit (U) of enzymatic activity was defined as ref [20, 24].

### Enzyme characterization assay

A pH range of 4.5 to 7.5 and a temperature range from 30 to 75 °C were employed to determine the optimal pH and temperature for AmRha, while the highest AmRha activity was defined as 100%. The purified enzyme AmRha was incubated at 45 °C in the pH range of 4.5 to 7.5 (50 mM sodium phosphate buffer) for 2 h to determine the pH stability. To determine the thermostability of the recombinant AmRha, the enzymatic activities of the samples after incubation at pH 5.5 for suitable durations at 45 °C, 55 °C and 65 °C, respectively, were measured. The enzymatic activity of the sample without preincubation was defined as 100%.

The sugar tolerance of AmRha was determined by adding 0–100 mM rhamnose. The effects of organic solvents on AmRha were determined by adding 0%, 2%, 5%, 10%, 15%, 20%, 30% and 40% of DMSO, alcohol or methanol. The activity of the sample without addition was defined as 100%.

To determine the kinetic constant of AmRha for *p*NPR hydrolysis, the initial reaction rates were investigated in pH 5.5 at 65 °C under various substrate concentrations (0.2, 0.4, 0.8, 1.0, 1.2 and 1.4 mM). The specific enzymatic activities of AmRha on various natural substrates (epimedin A, epimedin B, epimedin C, icariin, baohuoside I, myricetrin, hesperidin, rutin and naringin) were evaluated. The samples were assayed via HPLC. One unit of enzymatic activity on EFs was defined as the amount of enzyme that catalyzed 1 μmol of the substrate per min under the assay conditions.

### Preparation of icariin

A 100 μL reaction system contained 50 mM sodium phosphate buffer with different pH values (4.0, 4.5, 5.0, 5.5, 6.0, 6.5, 7.0, and 7.5), 25 g/L EFs mixture (Shaanxi, China), and different amount of AmRha (0.25, 0.5, 1, 2, 3 and 5 U/mL). The reactions were carried out at different temperatures (45 °C, 55 °C and 65 °C) for 4 h, and then terminated by the addition of 300 μL methanol. Samples were taken at 0, 0.25, 0.5, 1, 2, 3, 5 and 7 h, and then were analyzed via HPLC.

Conditions of icariin preparation from EFs mixture (Shaanxi, China) by engineered strain *GS115-AmRha* were investigated. The recombinant *GS115-AmRha* strain was cultured with BMGY medium according to the standard method. The initial medium pH was 6.0. All samples were detected by HPLC. 100 μL of each sample were taken and treated by 300 μL methanol. The conversion rate of icariin at the end of the reaction was compared. Firstly, two substrate addition modes were compared: (1) adding 50 g/L of the substrate along with the addition of methanol at the same time for induction and (2) adding 50 g/L of the substrate after expression of the enzyme for 1 d by methanol. Then the effects of different pH of phosphate buffer (4.0, 5.0, 6.0, 7.0 and 8.0) in BMGY medium on the biotransformation was investigated by the 6-h reaction in mode two. A special note is needed: the effect of pH on enzyme production by recombinant GS115-AmRha strain was examined in a preliminary pre-experiment, and the results showed that the highest recombinant expression was obtained when the pH was 6.0 (Additional file 1: Fig. S1). The effect of different yeast extract mass concentrations (0.5%, 1%, 1.5%, 2% and 2.5%) in BMGY medium on the biotransformation rate were investigated by the 6-h reaction in mode two.

The concentration of rhamnose during the biotransformation of EFs mixture (Shaanxi, China) into icariin by *GS115-AmRha* was monitored for determination of its effect on the disinhibition of product rhamnose. 100 g/L of EFs mixture (Shaanxi, China) was firstly added for a 12-h reaction in a manner of mode two mentioned. Then, the same amount of EFs mixture (Shaanxi, China) was continuingly added for another 12-h reaction. 100 μL of each sample was taken at intervals. Samples taken during the total 24-h reaction were detected by HPLC. The changes in rhamnose, epimedin C and icariin during the reaction were monitored.

### Preparation of baohuoside I from the EFs mixture

A 100 μL reaction system contained sodium phosphate buffer (50 mM) and 25 g/L EFs mixture (Jiangsu, China), terminated via the addition of 400 μL methanol and detected by HPLC.

The addition modes of recombinant AmRha and Dth3 were investigated firstly. The conversion rate of baohuoside I was compared in three enzyme addition modes. Mode one means that recombinant AmRha and Dth3 were added into the reaction system together for a 4-h reaction in pH 5.5 at 45 °C. Mode two means that recombinant AmRha was added into the system for a 2-h reaction before the 2-h reaction of Dth3 in pH 5.5 at 45 °C. Mode three means that recombinant Dth3 was added into the system for a 2-h reaction before the 2-h reaction of AmRha in pH 5.5 at 45 °C.

The optimal reaction pH of the biotransformation of EFs mixture (Jiangsu, China) to baohuoside I was compared under various pH values (4.0, 4.5, 5.0, 5.5, 6.0, 6.5, 7.0, and 7.5). The optimal enzyme dosages of AmRha and Dth3 were determined by adding AmRha to the reaction mixture to final concentrations of 0.25, 0.5, 1, 1.5, 2 and 2.5 U/mL for a 4-h incubation in pH 5.5 at 45 °C, with excess addition of Dth3. The optimal enzyme dosage of Dth3 was determined by adding Dth3 into the reaction mixture to final concentrations of 0.25, 0.5, 0.75, 1, 1.5, 2 and 2.5 U/mL for a 4-h incubation in pH 5.5 at 45 °C, with excess addition of AmRha.

The conversion rate of baohuoside I during the reaction process was monitored respectively, under different reaction temperatures (65 °C, 55 °C and 45 °C). The one-step hydrolysis mode I was adoped, in which the enzyme AmRha (1.5 U/mL dosage) and Dth3 (1.5 U/mL dosage) were added together. The changes of epimedin A, epimedin B, epimedin C, icariin and baohuoside I at 45 °C were monitored for determination of the reaction time course of cooperated biotransformation of EFs mixture (Jiangsu, China) into baohuoside I by AmRha and Dth3. All the samples were measured at 0–24 h time points.

### HPLC and Liquid chromatography-mass spectrometry (LC–MS) analysis

The EFs analysis was proceeded in an HPLC 1260 system (Agilent, USA) and used a C18 column (4.6 mm × 250 mm; i.d., 5 μm; S.No. USNH017518, USA). Distilled water (solvent A) and acetonitrile (solvent B) were used as the mobile phases followed by the method: a gradient elution of 20%-28% solvent B (0–35 min), 28–40% solvent B (35–40 min), 40–90% solvent B (40–50 min) and 90–20% solvent B (50–55 min). Icariin preparation was followed by the method: a gradient elution of 28% solvent B (0–20 min), 28–63% solvent B (20–25 min), 63–90% solvent B (25–30 min), and 90–28% solvent B (30–35 min). The injection volume was 20 μL for each sample. The flow rate was 1 mL/min. The detection was conducted by measuring the absorbance at 270 nm. The concentrations of the EFs were calculated according to the standard equations directly as ref [20].

The concentration of rhamnose analysis was proceeded in an HPLC 1200 system (Agilent, USA) and used a Bio-Rad Aminex HPX-87H column (7.8 mm × 300 mm, USA). 0.005 mol/L H_2_SO_4_ was used as the mobile phase. The flow rate was 0.6 mL/min. The injection volume was 10 μL for each sample. The concentration of rhamnose was determined by an external standard method using a differential refractive index detector (RID). The concentration of rhamnose was calculated according to the standard equations (*y* = 150132.61*x* + 661.24, *R*^2^ = 0.9999, in which *x* indicated the mass concentration of rhamnose and *y* indicated the corresponding peak area).

A triple-quadrupole tandem mass spectrometer (Agilent, USA) was used to perform the mass spectrometric experiment. The detection was performed in + ESI mode, the probe temperature was 400 °C, the probe voltage was 180.0 V, and the flow rate was 0.4 mL/min. The compounds were analyzed in full scan mode with N_2_ as the interface gas.

## Results and discussion

### Enzyme screening and bioinformatics analysis of AmRha

As reported, only two *α-*l*-*rhamnosidases (BtRha [14] from *Bacteroides thetaiotaomicron* VPI-5482 and AnRha [17] from *Aspergillus nidulans*) could transform epimedin C into icariin, while their limited efficiency (extremely large amount of enzyme dosage and low concentration of transformed substrate) obviously impedes their industrial application. Therefore, in order to screen for *α-*l*-*rhamnosidases with efficient and highly selective excision of the *α-*1,2*-*rhamnosidic bond between the double rhamnosyl groups of epimedin C, the amino acid sequences of the reported *α-*l*-*rhamnosidases BtRha and AnRha were used as templates for homology comparison in the NCBI database, and 9 strains from fungi and 8 strains from bacteria were screened. An evolutionary tree with high reliability was constructed by the adjacency method (Additional file 1: Fig. S2a). The *α-*l*-*rhamnosidase from *Aspergillus mulundensis* is from the same branch as the *α-*l*-*rhamnosidase AnRha from *Aspergillus nidulans*, and the sequences of the two have up to 81.6% similarity, which was affiliated with the glycoside hydrolase (GH) 78 family. It indicated that the enzyme AmRha may show similar substrate specificity with the reported enzyme AnRha. The BtRha from *Bacteroides thetaiotaomicron* VPI-5482 [14] belongs to the same branch as the *α-*l*-*rhamnosidases from *Dictyoglomus thermophilum* H-6-12 [25] and *Thermotoga petrophila* RKU-1 [26]. However, all three bacterial sources of *α-*l*-*rhamnosidases mentioned above have been studied. And the reported TpeRha [26] did not have absolute specificity for the *α-*1,2*-*rhamnosidic bond between the double rhamnosyl groups.

For further investigation, the amino acid sequences of AmRha and other *α-*l*-*rhamnosidase from GH78 families were compared. As shown in Additional file 1: Fig. S2b, *α-*l*-*rhamnosidase AmRha and AnRha shared the same conserved region. However, *α-*l*-*rhamnosidase of *Bacillus *sp. GL-1 [27] origin and *Streptomyces avermitilis* MA-4680 [28] origin has the same catalytical conserved region as AmRha, but their two-dimensional structures differ significantly.

In order to determine whether AmRha exhibits absolute specificity for the *α-*1,2*-*rhamnosidic bond and its substrate specificity differences from other *α-*l-rhamnosidases of the same GH family, it is necessary to obtain the enzyme protein and determine its enzymatic activity against the artificial substrate* p*NPR and other typical natural substrates containing a double rhamnosyl group. Therefore, we cloned and expressed the gene AmRha with *Komagataella phaffii* GS115 as the host. Through the full sequence analysis of the genomic cDNA of *Aspergillus mulundensis*, only one possible *α-*l-rhamnosidase gene *AmRha* was found (GenBank access No. XM_026751123.1). The total length of *α-*l*-*rhamnosidase gene *AmRha* is 2589 bp encoding 862 amino acids, the theoretical molecular mass is about 107.27 kDa, and the predicted isoelectric point pI is about 4.73.

**Fig. 2:**
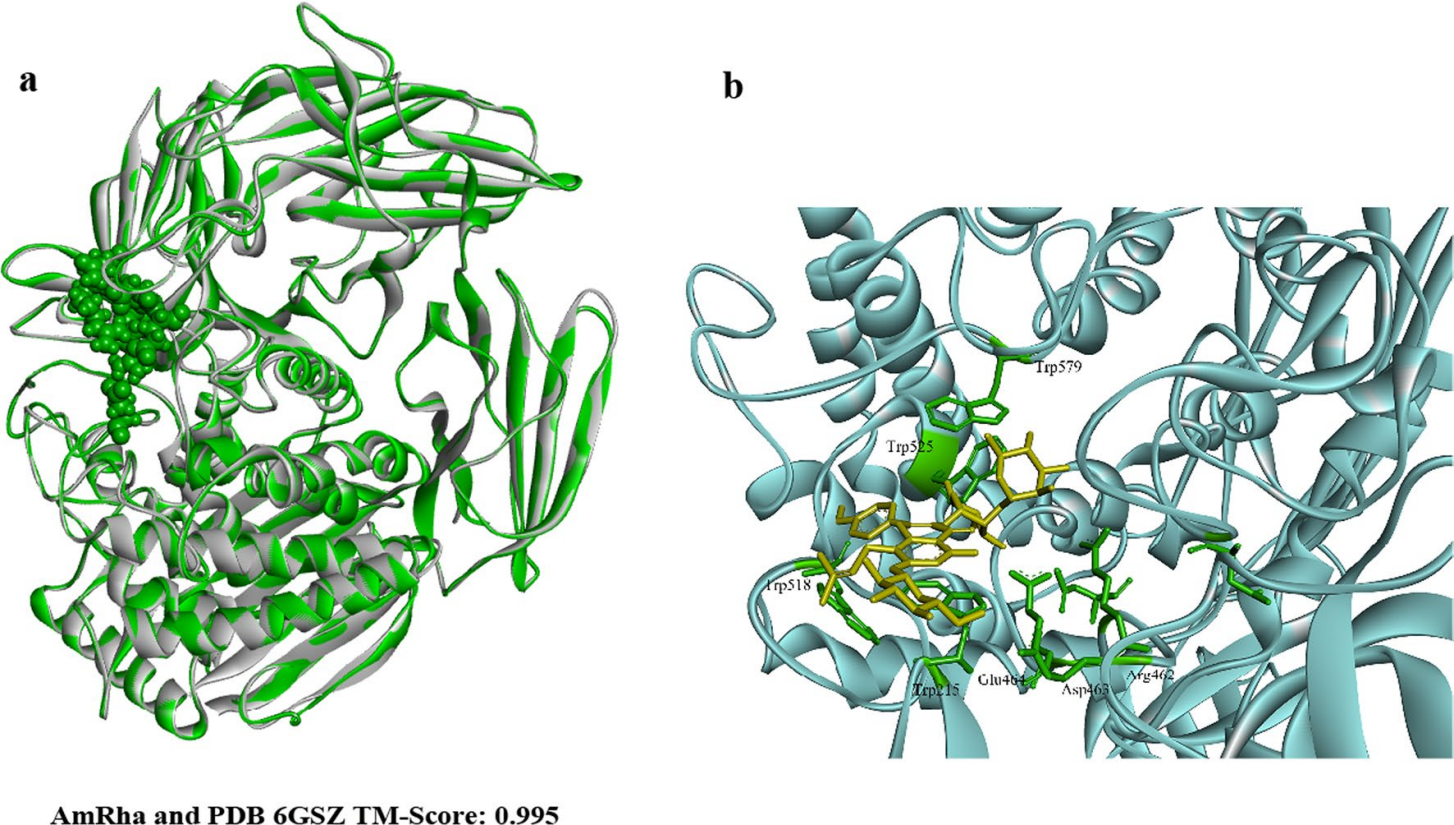
Enzyme screening and bioinformatics analysis of AmRha. **a** Comparison of ligand binding sites of AmRha with PDB 6GSZ. **b** Molecular docking of enzyme AmRha and epimedin C (Glu758, Trp579, Asp458, Glu464, Trp525, Arg462, Arg378, Trp215, Arg463, Trp518)

To date, 6 sources of *α-*l-rhamnosidases are known to have their crystal structures resolved, which IDs in the PDB database are 6I60, 6GSZ, 4XHC, 3W5M, 3CIH, and 2OKX, respectively [29, 30]. Among them, only AtRha (6GSZ) from *A. terreus* [31] had the highest similarity with AmRha at 67.1%, while the others were less than 30%. AmRha of I-TASSER modeling was built (Additional file 1: Fig. 2a) using the structure template of 6GSZ selected from the LOMETS threading programs with Z-score of 12.62, which implies good alignment. The C-score ([−5–2] range) can be used as one of the key convergence parameters of the structure assembly simulations, where a high C-score value indicates a model with a high confidence. In this paper, the C-scores were 2.0, which indicated that the final model predicted by I-TASSER was realistic. In addition, TM-score was 0.99 ± 0.03, RSMD was 3.6 ± 2.5 Å. To sum up, the simulated 3D structure of AmRha is plausible for subsequent bioinformatics analysis. Based on the 3D structure of AmRha, COACH software predicted the substrate binding sites of the enzyme, which were Asp458, Arg462, Glu464, Trp468, Asp471, Trp525, Phe576, Trp579, Phe694, His759. Furthermore, the key amino acid residues of the enzyme protein–substrate interaction were obtained by docking epimedin C containing a double l-rhamnose group with AmRha, which were Glu758, Trp579, Asp458, Glu464, Trp525, Arg462, Arg378, Trp215, Arg463, and Trp518 (Fig. 2b).

### Enzymatic purification and enzymatic properties of AmRha

#### Expression and purification of recombinant AmRha

**Fig. 3:**
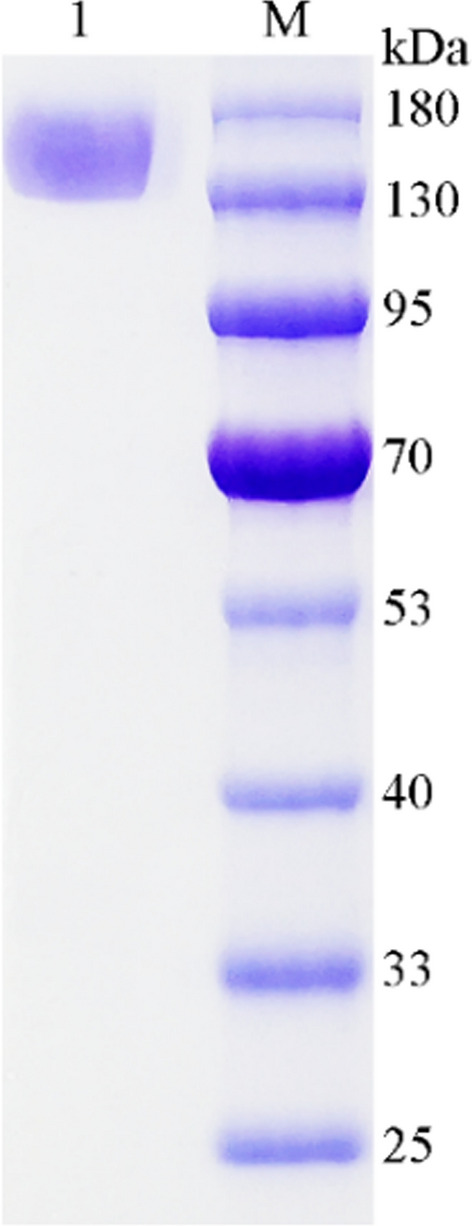
SDS-PAGE analysis of purified AmRha

AmRha was synthesized by the whole genes and attached to the *pPICZα*A vector. 23 positive transformants were screened by the YPDs plates with zeocin. The colonies were selected for induction expression. Finally, the recombinant strain with the highest enzyme activity was used to optimize the expression conditions of AmRha. Under the optimized conditions (peptone mass concentration of 2.5%, yeast extract mass concentration of 2.0%, pH 6.0 and 0.6% volume fraction methanol as inducer, data were not shown), the highest expressed enzyme activity of AmRha reached 571.04 U/mL at day 15, which was approximately 356-fold and 208-fold than that of the *α-*l-rhamnosidases from *Aspergillus oryzae* NL-1 [32] and *Aspergillus oryzae* KCGM 12698 [18], respectively, slightly lower than *α-*l-rhamnosidase AtRha from *Aspergillus terreus* [31]*.* After collecting the culture supernatant, the crude enzyme was obtained. The purified recombinant enzyme AmRha was obtained after gradient salting out, anion exchange chromatography (DEAE) and dialysis. SDS-PAGE was used to analyze the purified AmRha, where the recombinant AmRha has a single band at a position greater than 130 kDa (Fig. 3). The actual molecular mass is greater than the theoretical molecular mass, which may be resulted from the glycosylation of the protein expressed by *Komagataella phaffii* GS115 eukaryotic expression system [20]. The purified recombinant AmRha would be used for the subsequent determination of enzymatic properties.

#### Optimum reaction conditions and stability of AmRha

**Fig. 4:**
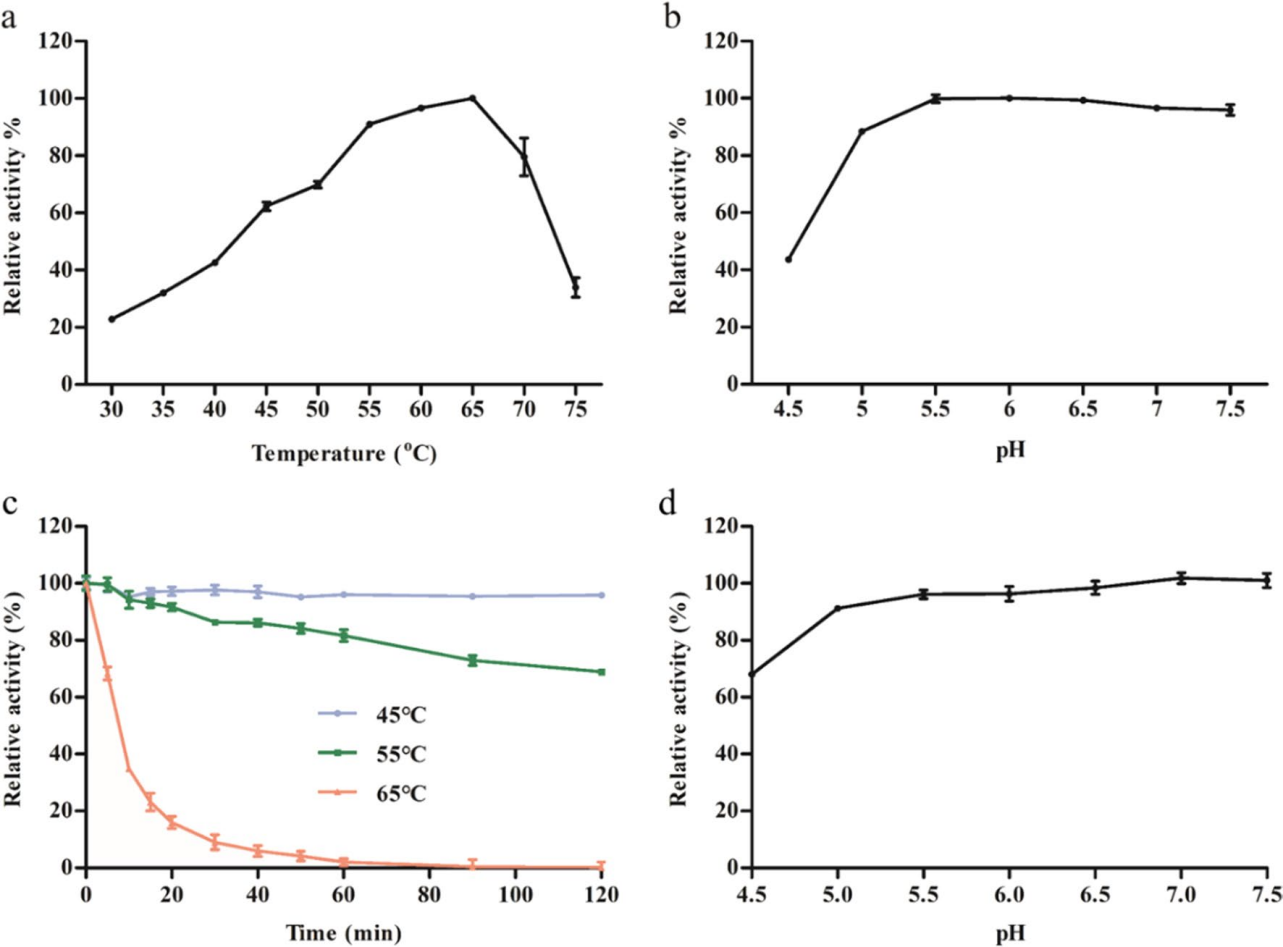
Optimum reaction conditions and stability of AmRha. **a** The optimum reaction temperature. **b** The optimum reaction pH. **c** Thermostability of AmRha. **d** pH stability of AmRha

The optimum reaction condition of AmRha was determined using artificial substrate *p*NPR. The optimum temperature of AmRha is 65 °C (Fig. 4a), which was similar to the *α-*l-rhamnosidases from *A. terreus* [31], *A. niger* [19] and *A. oryzae* NL-1 [32], but was significantly higher than the *α-*l-rhamnosidase from *Bacillus *sp. GL-1 [27] and *Sphingomonas *sp. R1 [20]. The residual enzyme activity of AmRha remains above 60% within the temperature range of 45–70 °C, indicating that AmRha has a wide application temperature range. The optimum reaction pH of AmRha is 5.5 (Fig. 4b), and the enzyme shows more than 80% relative enzyme activity in the range of pH 5.0–7.5. In addition, the recombinant enzyme may show excellent tolerance in pH range over 5.0, since its subtle change of enzyme activity over pH 5.0. In industrial applications, the enzyme is commonly incubated with the substrate at an appropriate temperature for a long time [26] or inconsistent with the physiological pH conditions [24], which required good enzyme thermostability and pH stability. The thermostability of AmRha is shown in Fig. 4c, which shows excellent thermostability at 45 °C and 55 °C. Over 60% of the residual enzyme activity was retained after incubating at 55 °C for 120 min, while the enzyme activity was with a subtle change after incubating at 45 °C for 120 min. Different pH environments would affect the stability of the protein structure, as shown in Fig. 4d, AmRha still retained more than 80% of the residual enzyme activity in the range of pH 5.0–7.5 at 45 °C for 1 h, which indicated that, AmRha could maintain a high level of enzyme catalytic ability and stability in the range of pH 5.0–7.5 and would suitable in many industrial applications.

#### Effects of metal ions, chemical reagents and sugars on enzyme activity of AmRha

As shown in Table 1, some metal ions (1 mM) showed weak activation effects on AmRha, such as Fe^2+^, Mn^2+^. While the enzyme activity of AmRha suddenly decreased from 83.9% to 9.3%, the Cu^2+^ concentration increased from 1 to 3 mM. Additionally, Hg^2+^ could inhibit the enzyme activity of AmRha strongly. The addition of chelating agent EDTA did not show any significant effect on the activity of AmRha. Therefore, it indicated that metal ions do not play a role in the catalysis and structure stability of the enzyme [15].

**Table 1:** Effects of metal ions on the activity of AmRha

Metal ions	Residual enzyme activity %	
	1 mmol/L	3 mmol/L
Control	100	100
Fe^3+^	92.65	112.78
Ni^2+^	98.98	99.80
Na^+^	98.78	98.58
Sr^2+^	98.37	99.19
Ca^2+^	98.98	98.38
Cu^2+^	83.88	9.33
Li^+^	100.00	99.80
Co^2+^	100.20	101.83
Zn^2+^	91.63	87.02
Mn^2+^	106.33	106.29
Mg^2+^	98.57	97.97
Ba^2+^	98.57	96.35
K^+^	99.80	100.20
Hg^+^	0.61	1.01
EDTA	101.02	98.99
Al^3+^	100.61	93.71
NH^4+^	102.45	100.81
Fe^2+^	106.12	124.95

**Fig. 5:**
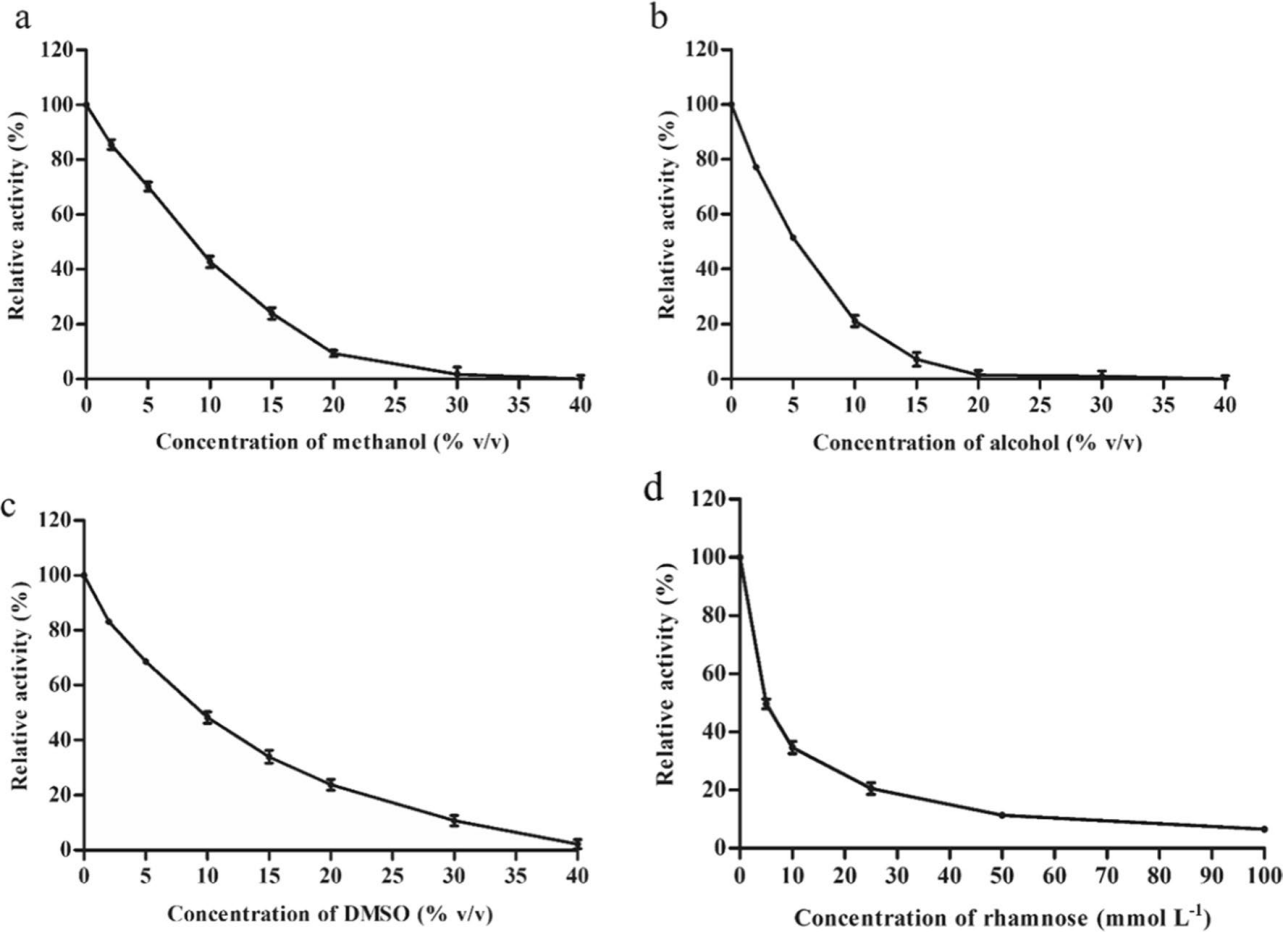
Organic solvent and rhamnose tolerance of AmRha. **a** Methanol. **b** Alcohol. **c** DMSO. **d** Rhamnose

In industrial applications, most natural products are poorly soluble in water or water-insoluble [33]. It is commonly used for the addition of proper organic solvents to facilitate the dissolution of substrates, such as methanol, ethanol, dimethyl sulfoxide (DMSO), et al. However, some organic solvents may destroy the hydration layer on the protein surface. Additionally, a high concentration of polar organic solvent could directly denature the enzyme protein [33]. Therefore, determining the organic tolerances of the target enzyme could support the appropriate amount of organic solvent in industrial applications. As shown in Fig. 5, the tolerance of AmRha to DMSO was better than that of methanol and ethanol. The tolerance of AmRha to DMSO and methanol showed a similar trend under the concentration of less than 10% organic solvents, where more than 50% of the residual enzymatic activity was maintained. Additionally, over 50% of the residual enzyme activity was maintained with the addition of less than 5% ethanol.

Feedback inhibition is a familiar phenomenon in the reaction of glycosidase, where the product sugar could compete with the binding sites of the enzyme with the substrate [24, 34]. This would lead to the inhibition of the catalytic efficiency of the enzyme to the substrate. Therefore, the sugar tolerance of target glycosidase deeply influenced its catalytic efficiency under higher concentrations of substrate [34, 35]. In this investigation, different concentrations of l*-*rhamnose (0–100 mM) were added, with *p*NPR as the substrate, to test the l*-*rhamnose tolerance of AmRha. As shown in Fig. 5d, the remaining enzyme activity of recombinant AmRha is 49.7% with the addition of 5 mM l*-*rhamnose.

### Substrate specificity and kinetic parameters of AmRha

For determining the types of glycosides that AmRha could hydrolyze the artificial substrates* p*NPR, *p*NPG, *p*NPX, *p*NPAraf and *p*NPArap were selected as the substrates. As shown in Table 2, the recombinant AmRha only has specific activity on the l*-*rhamnose group but not on other glycosides, and its specific enzyme activity to *p*NPR was 434 U/mg. The *K*_*m*_, *V*_*max*_, *k*_*cat*_ and *k*_*cat*_*/K*_*m*_ values of recombinant AmRha to *p*NPR were 0.30 mM, 503.61 μmol/min·mg, 900.34 s^−1^ and 2969.27 s^−1^ mM^−1^, respectively. The calculated *K*_m_ value for AmRha was lower than the *α-*l*-*rhamnosidase from *A. terreus* [31], *A. ternaria *sp. L1 [36] and *A. oryzae* NL-1 [32], and the *k*_*cat*_ value for AmRha was greater than the *α-*l*-*rhamnosidases above, which indicated that the recombinant AmRha was significantly more capable of catalyzing the hydrolysis of rhamnosidic bonds.

**Table 2:** Substrate specificity of AmRha

Substrate	Specific activity U/mg	Bond type
*p*NPR	434.00	-α-1-Rha
*p*NPG	–	-α-1-Glc
*p*NPX	–	-α-1-Xyl
*p*NPAraf	–	-α-1-Araf
*p*NPArap	–	-α-1-Arap
Epimedin A	–	-α-1-Rha-Glc
Epimedin B	–	-α-1-Rha-Xyl
Epimedin C	18.23	-α-1-Rha**-α-1,2-**Rha
Icariin	–	-α-1-Rha
Baohuoside I	–	-α-1-Rha
Myricetrin	–	-α-1-Rha
Hesperidin	19.71	-Glc**-α-1,6-**Rha
Rutin	47.91	-Glc**-α-1,6-**Rha
Naringin	534.79	-Glc**-α-1,2-**Rha

Then, we further explored the type of *α-*rhamnoside bond of some natural substrates that AmRha could hydrolyze, such as epimedin C (*-α-*1*-*Rha*-α-*1,2*-*Rha), icariin (*-α-*1*-*Rha), hesperidin (*-*Glc*-α-*1,6*-*Rha), rutin (*-*Glc*-α*-1,6*-*rha), naringin (*-*Glc*-α-1,2-*Rha) and myricetin (*-α-*1*-*Rha). A systematic study of them is very vital for the guidance of *α-*l*-*rhamnosidase in biocatalytic conversion. As shown in Table 2, AmRha showed enzyme activity on naringin, rutin and hesperidin with the specific enzyme activities of 534.79, 47.91 and 19.71 U/mg, respectively. It indicated that AmRha could act on *α-*1,2*-*rhamnoside and *α-1,6-*rhamnoside bonds directly connected with glucose.

The specific enzyme activity of AmRha on the natural substrate epimedin C is 18.23 U/mg. Interestingly, AmRha could only remove the outer l*-*rhamnose group of epimedin C at the C-3 position to convert into icariin. The inner l*-*rhamnose group of epimedin C linked by *α-*1*-*rhamnoside bond could not be removed by recombinant AmRha, which indicated that AmRha could only transform the substrate epimedin C into icariin. Additionally, the product icariin could not be further hydrolyzed into icariside I. Till now, only two *α-*l*-*rhamnosidase were reported, both of which inhibited enzyme activity. The reaction kinetic parameters of recombinant AmRha were tested using epimedin C as the substrate. The *K*_*m*_, *V*_*max*_, *k*_*cat*_ and *k*_*cat*_*/K*_*m*_ values of recombinant AmRha to epimedin C were 6.96 mM, 20.76 μmol/min·mg, 37.11 s^−1^ and 5.33 s^−1^ mM^−1^, respectively, which showed the highest catalytic efficiency in transforming epimedin C into icariin.

### Evaluation of AmRha on icariin preparation in vitro

Epimedin C is the most abundant flavonoid in the EFs mixture from Shaanxi province (China), followed by icariin and then baohuoside I [37]. Therefore, this type of EF would be suitable as substrate in icariin preparation by selective hydrolysis of the most abundant epimedin C. As investigated in substrate specificity, AmRha could strictly disconnect the *α-*1,2-rhamnoside bond between two l*-*rhamnose groups with removement of the outer l-rhamnose group but not the outer one. Therefore, AmRha would be an excellent solution to the difficulty in selective hydrolysis of epimedin C in EFs mixture (Shaanxi, China) for product icariin. Epimedin C just could be transformed into only product icariin. In order to verify the ability of AmRha on transforming epimedin C into icariin, 5 mM and 10 mM of epimedin C were experimented in consideration of the enzyme characterization investigated above. Finally, 5 mM of epimedin C was almost completely converted into icariin with a molar conversion rate of 92.0% by 2.5 U/mL AmRha at pH 6.0 under 45 °C for 6 h (data were not shown). Additionally, 10 mM of epimedin C was converted into icariin with a molar conversion of 91.1% by 5 U/mL AmRha under the same conditions (data were not shown). It indicated the great potential of AmRha on selective hydrolysis of epimedin C in EFs mixture (Shaanxi, China), which would play an important role in producing icariin or baohuoside I under high substrate concentrations.

**Fig. 6:**
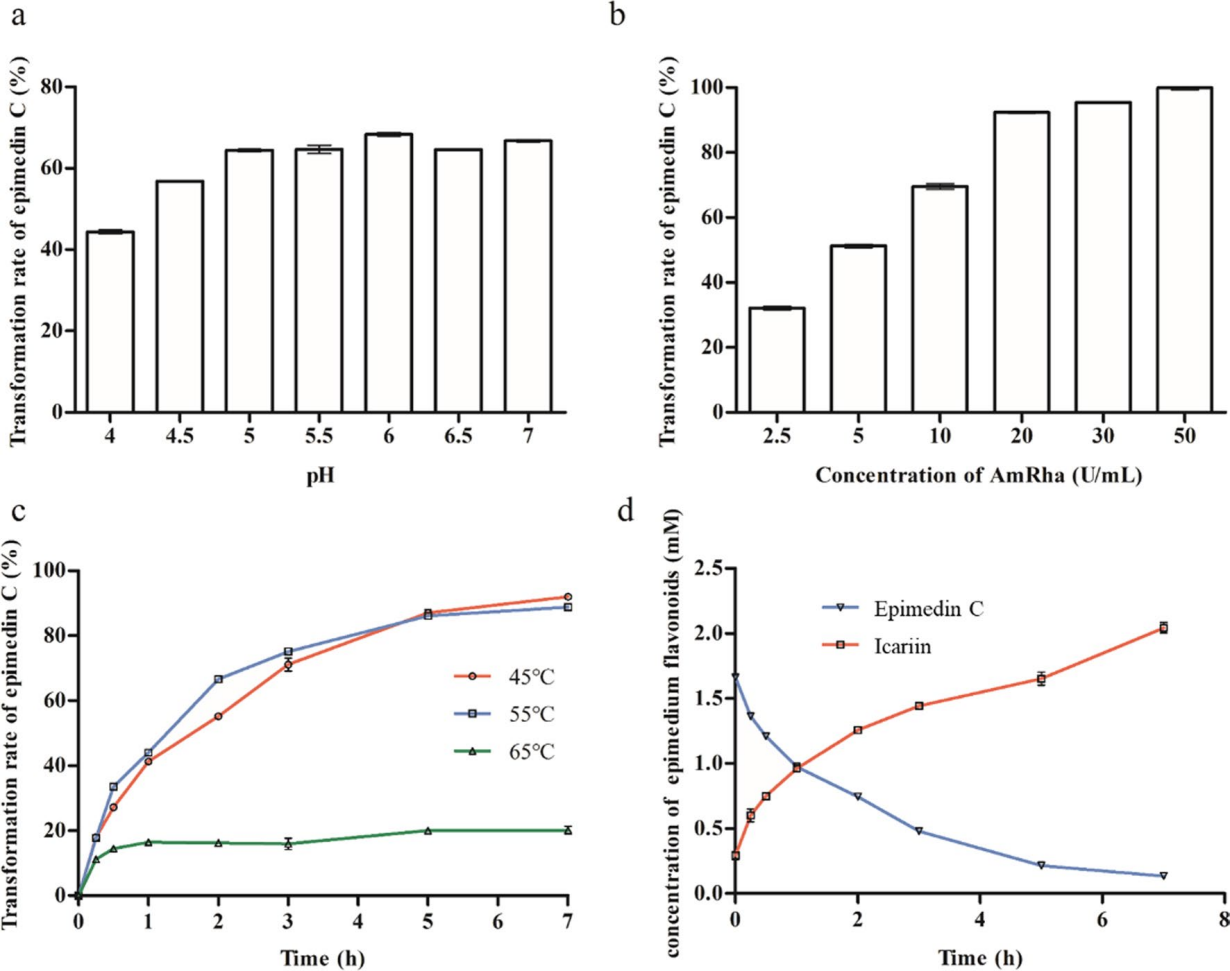
Optimization of in vitro transformation of EFs into icariin by AmRha. **a** Optimization of pH. **b** Optimization of AmRha concentration. **c** Effect of temperature. **d** Reaction time course

The in vitro transformation experiment of selective hydrolysis of epimedin C in EFs mixture (Shaanxi, China) for product icariin by AmRha was explored. As shown in Fig. 6a, the optimum reaction pH for the conversion of epimedin C in in EFs mixture (Shaanxi, China) was pH 6.0. AmRha was added into the reaction system individually with different ultimate concentrations, following the reaction in pH 6.0 at 45 °C for 2 h, for determining the effect of the amount of AmRha on the conversion rate of epimedin C in EFs mixture (Shaanxi, China). As shown in Fig. 6b, almost all the epimedin C in 25 g/L in EFs mixture (Shaanxi, China) from Shaanxi province (China) was transformed into icariin by 20 U/mL of recombinant AmRha with the molar conversion rate of 92.3%. In order to evaluate the effect of reaction temperature on the conversion rate of epimedin C in EFs mixture (Shaanxi, China) during the reaction, changes in epimedin C conversion rate at different temperatures were monitored throughout the reaction (Fig. 6c). The epimedin C conversion rate of reaction at 65 °C after 1 h by AmRha tended to be stable and lower than that at 55 °C and 45 °C, since the recombinant AmRha in the reaction system was almost completely inactivated in that time. The epimedin C conversion rate at 55 °C was the highest, then followed by that at 45 °C and 65 °C, within the first 5 h. The trend of epimedin C conversion rate at 55 °C was similar to that at 45 °C. The catalytic efficiency at 55 °C is slightly higher than 45 °C due to the higher mass transfer rate of AmRha at 55 °C than that at 45 °C [27]. However, the epimedin C conversion rate at 45 °C exceeded that at 55 °C, 5 h later, since AmRha showed excellent stability for a longer time at 45 °C than 55 °C. This was consistent with the trend of temperature stability of recombinant AmRha shown in Fig. 4c. Finally, epimedin C in 25 g/L of EFs mixture (Shaanxi, China) could be almost completely transformed with a molar conversion rate of 92.0% by only 10 U/mL of recombinant AmRha under reaction at 45 °C for 7 h. Therefore, in some reactions of natural products catalyzed by enzymes in vitro, reducing the amount of enzyme and prolonging the reaction time at lower temperature might obtain similar result as that in the short-time reaction with more enzyme at higher temperature [15]. The changes of epimedin C and icariin throughout the reaction at 45 °C were monitored. As shown in Fig. 6d, the contents of epimedin C and icariin in the reaction system were 1.66 mM and 0.29 mM, respectively, at the beginning of the reaction. Epimedin C decreased gradually following the increase of icariin. Finally, the content of icariin in the reaction system was 2.04 mM with almost all epimedin C being transformed.

It is a pity that the substrate concentration in reactions above could hardly increase, since the efficiency of the in vitro transformation experiment on selective hydrolysis of epimedin C in EFs mixture (Shaanxi, China) for product icariin by AmRha was inhibited by the product l*-*rhamnose, due to the poor l*-*rhamnose tolerance of AmRha (Fig. 5d). l*-*rhamnose, also named 6-deoxy*-*l*-*mannose, is a common substance in plant polysaccharides, plant gums and glycosides, bacterial polysaccharides and so on. As reported, many microorganisms could use l*-*rhamnose as a carbon source [5]. In addition, phosphorylation and non-phosphorylation pathways are the two main metabolic pathways of l*-*rhamnose in organisms, among which the non-phosphorylation pathway exists in fungi and a few bacteria. The non-phosphorylation metabolic pathway has been wholly elucidated in *Scheffersomyces stipites* [38] and *Debaryomyceshansenii* [39]. l*-*rhamnose can be transformed into l*-*rhamnosin-l*-*lactone, l*-*rhamnoic acid and 3,6*-*dideoxy*-*l*-*chiogulose respectively, through the enzymes encoded by genes that *RHA1, LRA2, LRA3* and *LRA* [39]. The last two products, pyruvate and l*-*lactaldehyde, finally enter the central metabolic pathway. Another study showed that *Komagataella phaffii* could grow normally in the culture medium with l*-*rhamnose as the only carbon source [15]. Based on that, the substrate was added directly into the medium that the engineered *Komagataella phaffii* cells grew after the exocrine expression of the target enzyme induced by methanol. We assumed that the product l*-*rhamnose in the reaction process might be utilized through the living *Komagataella phaffii* cells, relieving the feedback inhibition of product l*-*rhamnose. In this paper, the engineered strain *GS115-AmRha* might be a feasible method for releasing the feedback inhibition of product l*-*rhamnose, since the engineered cell might utilize the product l*-*rhamnose as a carbon resource [28, 29].

### Evaluation of AmRha icariin preparation by engineered *Komagataella phaffii* AmRha-GS115 strain

First of all, it was necessary to confirm whether the substrate added directly into the culture medium with engineered *Komagataella phaffii* cells growing could be transformed into icariin or not. Two different substrate addition modes were explored. In addition, we monitored the OD_600_ of the *Komagataella phaffii* GS115 cells along with the substrate and product concentrations, the results showed that the growth period of the *Komagataella phaffii* GS115 cells was not inhibited during the transformation section, but showed the same growth trend as without the substrate (data were not shown).

**Fig. 7:**
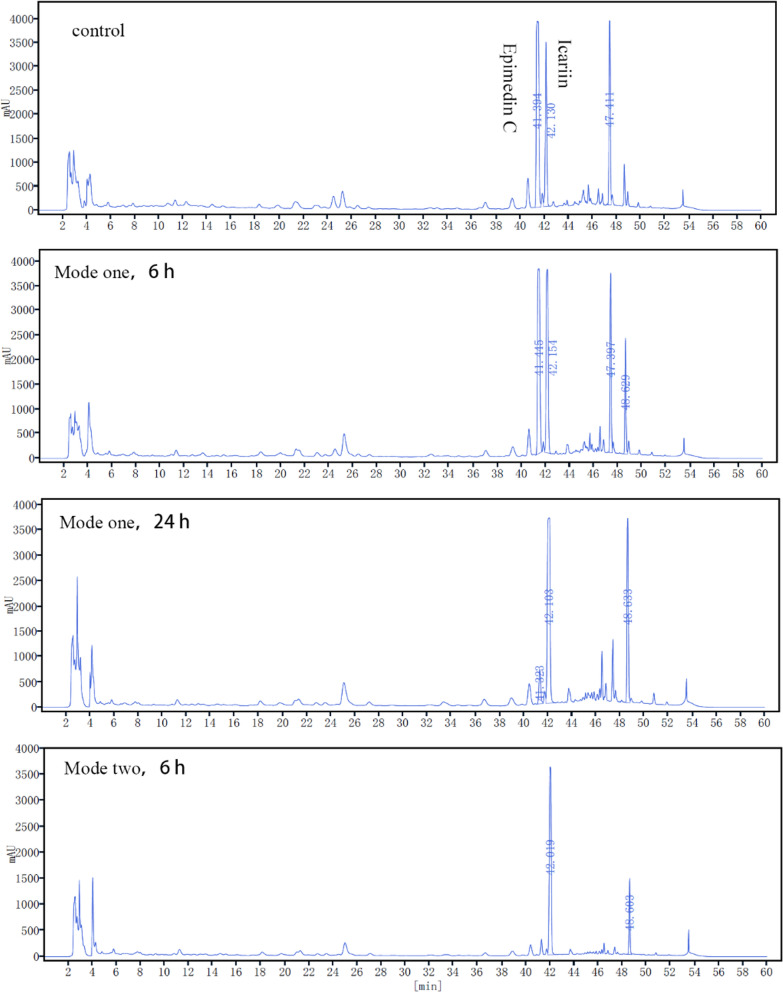
Optimization of substrate addition method for transformation of epimedin C by GS115-AmRha engineering strain

As shown in Fig. 7, in mode I, where the substrate was added while the initial addition of methanol, a large amount of substrate epimedin C was still retained 6 h after the addition of the substrate, and epimedin C was not completely transformed after 24 h by the addition of the substrate. In mode II, where the substrate was added for a reaction after the exocrine expression of the target enzyme induced by methanol for one day, epimedin C in the EF was completely transformed into icariin after 6 h of reaction. Therefore, in this study, mode II was selected for further investigations. Secondly, the medium conditions directly influence the growth of the engineered *Komagataella phaffii* cells, the expression of the target enzyme and the transformation efficiency of a substrate. Therefore, the pH of the phosphate buffer of the medium and the concentration of yeast extract were optimized. The conversion efficiency was higher when the pH of phosphate buffer was 6.0 and the concentration of yeast extract was 0.5%.

**Fig. 8:**
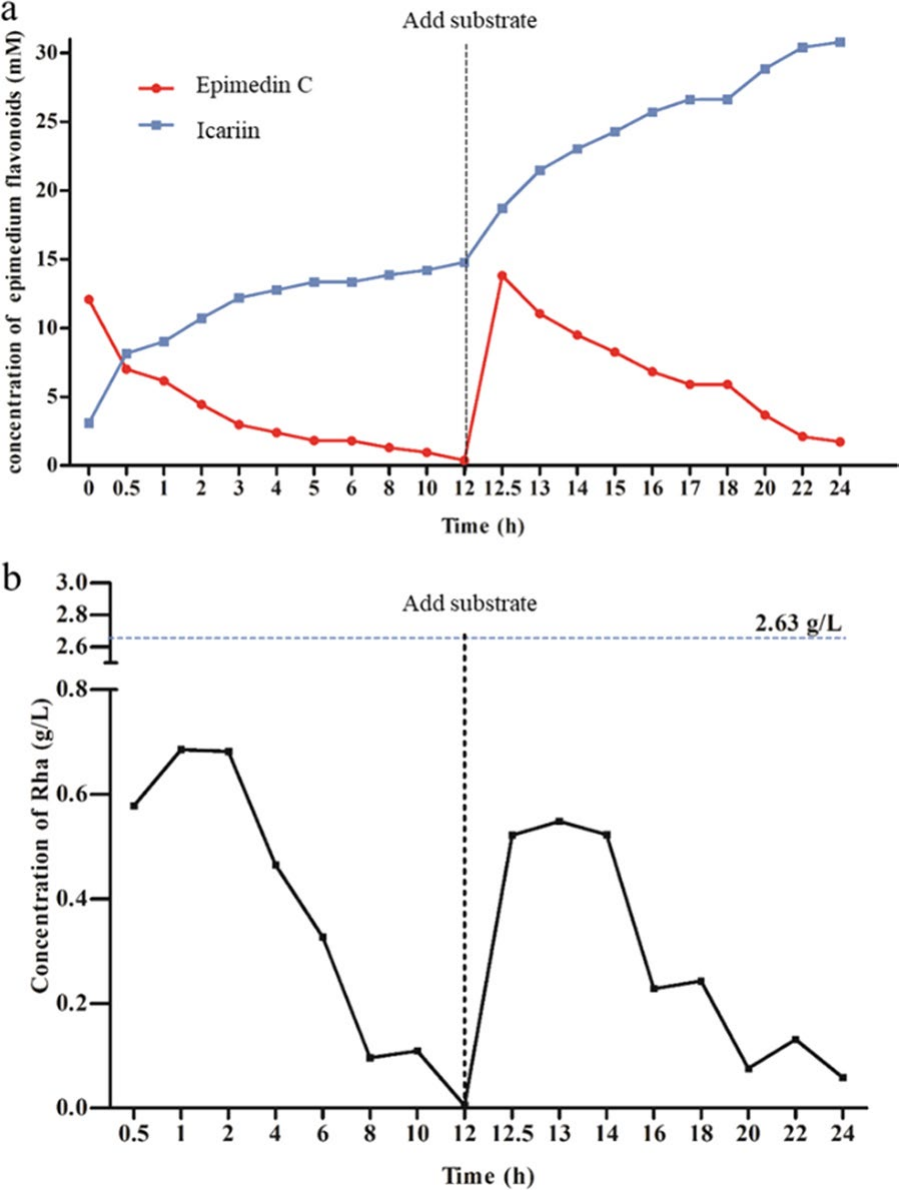
Time course of transformation of EFs (Shaanxi, China) by GS115-AmRha strain and the changes of rhamnose content in transformation system

Finally, the assumption that the product l*-*rhamnose in the reaction process might be utilized through the living *Komagataella phaffii* cells, was investigated for abolishing the product l*-*rhamnose inhibition. The optimized medium conditions were adopted and the engineered *Komagataella phaffii* strain was cultured according to the standard method. The enzyme activity of recombinant AmRha in the culture medium was about 40 U/mL after the expression of AmRha induced by methanol in 1 d. 100 g/L of EFs mixture (Shaanxi, China), containing abundant epimedin C, was then added to the culture medium with new methanol feeding. With the increase of product icariin, the content of epimedin C decreased gradually (Fig. 8a). The epimedin C in the substrate was almost completely transformed after a 12-h reaction. Then the same amount of substrate above was continuously added into the BMGY medium. The content of icariin in the culture medium increased continuously with the decrease of epimedin C, which showed a similar trend as the first-time reaction. The changes of l*-*rhamnose through the reaction process were monitored by HPLC.

As shown in Fig. 8b, the detected l*-*rhamnose content was 2.63 g/L when 100 g/L substrate was completely transformed by purified AmRha in vivo. While, the content of l*-*rhamnose remained below 0.7 g/L in the engineered *Komagataella phaffii* reaction system not only during the process of the first-time reaction of 100 g/L substrate, but the process of the second-time reaction of another addition of the same substrate. The trends of l*-*rhamnose content were the same in the two-reaction process. The content of l*-*rhamnose increased slightly within 1 h after the addition of substrate, which might result from the faster conversion rate of epimedin C than l*-*rhamnose utilizing rate of engineered *Komagataella phaffii* due to the high concentration of epimedin C after the addition of substrate. However, the utilization of l*-*rhamnose by engineering *Komagataella phaffii* was a dynamic process. The l*-*rhamnose content always decreased after the initial 1-h reaction. Finally, the product l*-*rhamnose could almost be utilized at the end of the reaction. It indicated that transformation by engineering *Komagataella phaffii* could realize the assumption for reliving the feedback inhibition under high substrate concentration, compared with the in vitro transformation by purified enzyme.

### Cooperate biotransformation of the total EFs into Baohuoside I by two glycosidases AmRha and Dth3

Baohuoside I is one of the most effective components of *Epimedium Folium* flavonoids [1, 2], which exhibited similar pharmacological activities with those of icariin, it is worth noting that baohuoside I shows the most significant effect on treatment of osteoporosis in contrast to other *Epimedium Folium* flavonoids [6–8]. In the total EFs, which contains more flavonoid fractions, such as epimedin A/B/C, icariin, and baohuoside I. Considering conventional acid-mediated hydrolysis of flavonoid glycosides lacking in regioselectivity and concern for environment, enzymatic bioconversion of epimedin A/B/C and icariin to baohuoside I by enzymes is expected to make up for the lacks of acid hydrolysis as a preparation method for selective degradation, environmental friendly and efficient conversion [40]. The preparation of baohuoside I from icariin by enzymatic hydrolysis or biotransformation has been widely studied. The substrate commonly used was the purified icariin or only the icariin ingredient in the total EFs. The purification of icariin would increase the cost of the substrate. It is the waste for transforming icariin ignoring other main ingredient in the total EFs into baohuoside I. Meanwhile, the enzyme used for the preparation of baohuoside I from icariin was only glucohydrolase. As analyzed, the key point on hydrolysing epimedin A/B/C and icariin into baohuoside I was the strict hydrolysis of to *α-*1,2*-*rhamnosidic bond other than *α-*1*-*rhamnosidic bond.

The challenge in the transformation of all the main ingredients of the total EFs was the screening of effective *α-*l*-*rhamnosidase with special specificity that could efficiently hydrolyze the *α-*1,2*-*rhamnoside bond between two rhamnose. That is to say the *α-*l*-*rhamnosidase could only transform the epimedin C into icariin without further hydrolysis of the inner rhamnosyl at C-3 of icariin. As mentioned, only two enzymes were reported [14, 17]. In this work, we found a novel *α-*l*-*rhamnosidase with this special specificity and the highest catalytic efficiency. In addition, it was applied in the biotransformation of the main ingredients of the total EFs into baohuoside I. The specific transformation process is shown in Fig. 1, which requires the participation of a multifunctional glycoside hydrolase with glucosidase and *β-*xylosidase activities, in addition to AmRha, which is capable of efficient and highly specific excision of the rhamnose base. Thus, the difficulty of converting the multi-component flavonoids in the total EFs into the product baohuoside I was solved. Therefore, the recombinant AmRha and the reported glucosidase Dth3 with *β-*xylosidase activity were adopted in preparation of baohuoside I from the total EFs (Fig. 1). The *α-*l*-*rhamnosidase AmRha could only transform epimedin C into icariin, while the glycosidase Dth3 could hydrolyze epimedin A, epimedin B and icariin into the product baohuoside I [15], after analyzing the structures of 4 major flavonoids in the total EFs. The assumption was investigated and the reaction conditions of cooperated biotransformation of the total EFs into baohuoside I by recombinant AmRha and Dth3 were optimized.

**Fig. 9:**
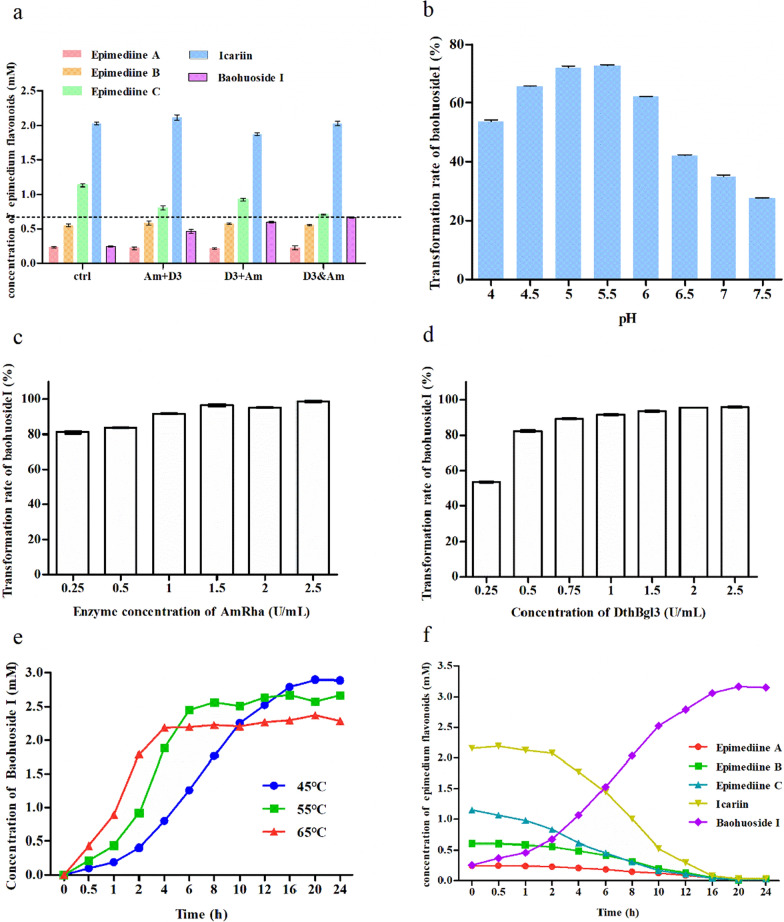
Optimization of conditions for preparation of baohuoside I by AmRha&Dth3 from EFs (Jiangsu, China). **a** Enzyme adding method. **b** Optimization of reaction pH. **c** Optimization the amount of recombinant AmRha. **d** Optimization the amount of recombinant Dth3. **e** The effect of reaction temperature on the production of baohuoside I. **f** Monitoring the reaction process

The addition modes of recombinant AmRha and Dth3 were explored firstly. The conversion rate of baohuoside I was the highest when recombinant AmRha and Dth3 were added together in the reaction system (Fig. 9a). And this addition mode was adopted for subsequent investigations. As shown in Fig. 9b, the conversion rate of baohuoside I was the highest under pH 5.5. Then, the effect of concentrations of recombinant AmRha and Dth3 on the conversion rate was determined. As shown in Fig. 9c, d, 1.5 U/mL of Dth3 and 1.5 U/mL of AmRha were enough for almost complete biotransformation of 25 g/L total EFs (Lianyungang, China) into baohuoside I under 45 °C for 4 h.

As reported, the recombinant Dth3 from thermophilic bacteria possesses an optimum reaction temperature of 85 °C [40], while the recombinant AmRha showed the highest catalysis efficiency at 65 °C. It indicated a certain gap between the optimum reaction temperatures of the two enzymes and the necessity for determining the effect of temperature on their transformation rate of four flavonoids (epimedin A/B/C and icariin) in the total EFs into baohuoside I. The temperature range was selected in view of the stability of recombinant AmRha, since the key in baohuoside I preparation from the total EFs was the complete transformation of epimedin C into icariin by AmRha. The recombinant Dth3 could be in a stable state for a long-time reaction, although it could not reach the greatest catalytic efficiency due to the reaction temperature lower than 85 °C. Therefore, the strategy (long-time reaction under low temperature) might be a solution for the low enzyme activity of the recombinant Dth3. Although the low reaction temperature reduces the catalytic efficiency of recombinant enzyme Dth3 to a certain extent, it can make the enzyme always maintain a stable state of catalytic activity in a long reaction time. Therefore, the reaction strategy of low temperature and a long time could compensate for the low catalytic efficiency of recombinant enzyme Dth3 due to low reaction temperature.

The conversion rate of baohuoside I during the reaction process was monitored respectively, under different reaction temperatures, for verifying the assumption above. As shown in Fig. 9e, the conversion rate of baohuoside I was the highest at 65 °C within the first 4 h of the reaction, followed by that at 55 °C and then at 45 °C. In addition, the feedback inhibition of product l*-*rhamnose of recombinant AmRha weakened its catalytic efficiency to a certain extent. The content of baohuoside I increased gradually during the process of the 24-h reaction at 45 °C, although its initial conversion rate of baohuoside I was lower than that at 55 °C and 65 °C. Additionally, the content of baohuoside I was the highest after reaction for 20 h. The final conversion rate of baohuoside I at 45 °C was the highest, where almost all the four major flavonoids (epimedin A/B/C and icariin) transformed into baohuoside I, which proved that the recombinant Dth3 from thermophilic bacteria could work well throughout long-time reaction at the low reaction temperature.

The changes of epimedin A/B/C, icariin and baohuoside I at 45 °C were monitored for determination of the reaction time course of cooperated biotransformation of the total EFs into baohuoside I by AmRha and Dth3. Epimedin A/B/C and icariin decreased gradually and completely transformed in 20 h (Fig. 8f). Epimedin C was converted into icariin by AmRha from the beginning of the reaction. However, the content of icariin was not increased from beginning to end, due to the higher transformation rate of icariin into baohuoside I by Dth3 than that of epimedin C into icariin by AmRha throughout the reaction. From the beginning of the reaction, the content of baohuoside I has been increasing. The growth of baohuoside I slowed down after reaction for 10 h, due to the feedback inhibition of the accumulation of product sugars. Finally, the content of baohuoside I was the highest after reaction for 20 h.

## Conclusions

To sum up, a novel GH 78 *α-*l*-*rhamnosidase AmRha from *Aspergillus mulundensis,* with the strict substrate specificity on the *α-*1,2*-*rhamnoside bond between two rhamnoses, was characterized. It could strictly hydrolyze epimedin C into icariin. The high-level expression was established in the engineered *Komagataella phaffii* GS115 with the activity of 571.04 U/mL. Then, the system for icariin transformation by recombinant AmRha in vitro and engineered *Komagataella phaffii* AmRha-GS115 strain was established. By the addition of 20 U/mL recombinant AmRha, epimedin C in 25 g/L EFs mixture (Shaanxi, China) was biotransformed into icariin with a molar conversion rate of 92.3% after 4 h under pH 6.0, 45 °C, in vitro. To further address the feedback inhibition of AmRha by l*-*rhamnose under high substrate concentration and improve biotransformation efficiency, the bioconversion was carried out in the engineered *Komagataella phaffii* AmRha-GS115 strain, in which the feedback inhibition of l*-*rhamnose was largely removed and could enable the concentration of raw EFs to improve fivefold.

In addition, we first reported that epimedins A, epimedins B, epimedins C and icariin in the total EFs can convert efficiently to baohuoside I by a collaborative of AmRha and multifunctional glycosidase Dth3. Under the reaction condition of 25 g/L total EFs (Lianyungang, China), 1.5 U/mL Dth3 and 1.5 U/mL AmRha, reacting in pH 5.5 at 45 °C for 24 h, the content of baohuoside I was the highest. The engineered *Komagataella phaffii* AmRha-GS115 strain revealed the promising potential in the industrial production of high-value product icariin and provides a new insight into the preparation of icariin and baohuoside I from cheap raw EFs.

## Supplementary Information

**Additional file 1:** **Fig. S1. **The effect of concentration of methanol, pH, concentration of peptone and concentration of yeast extract on enzyme production by recombinant GS115-AmRha strain. **Fig. S2.** Enzyme screening and bioinformatics analysis of AmRha.

## Data Availability

Data are available on request from the authors.
