# Supplementary material for: Efficient production of icariin and baohuoside I from Epimedium Folium flavonoids by fungal α-l-rhamnosidase hydrolysing regioselectively the terminal rhamnose of epimedin C
Source: Biotechnol Biofuels Bioprod. 2023 Jun 30;16:107. doi: 10.1186/s13068-023-02348-6 (PMC10311867; doi:10.1186/s13068-023-02348-6)
Supplement: Supplementary file 1 — Additional file 1: Fig. S1. The effect of concentration of methanol, pH, concentration of peptone and concentration of yeast extract on enzyme production by recombinant GS115-AmRha strain. Fig. S2. Enzyme screening and bioinformatics analysis of AmRha. [file 13068_2023_2348_MOESM1_ESM.docx]

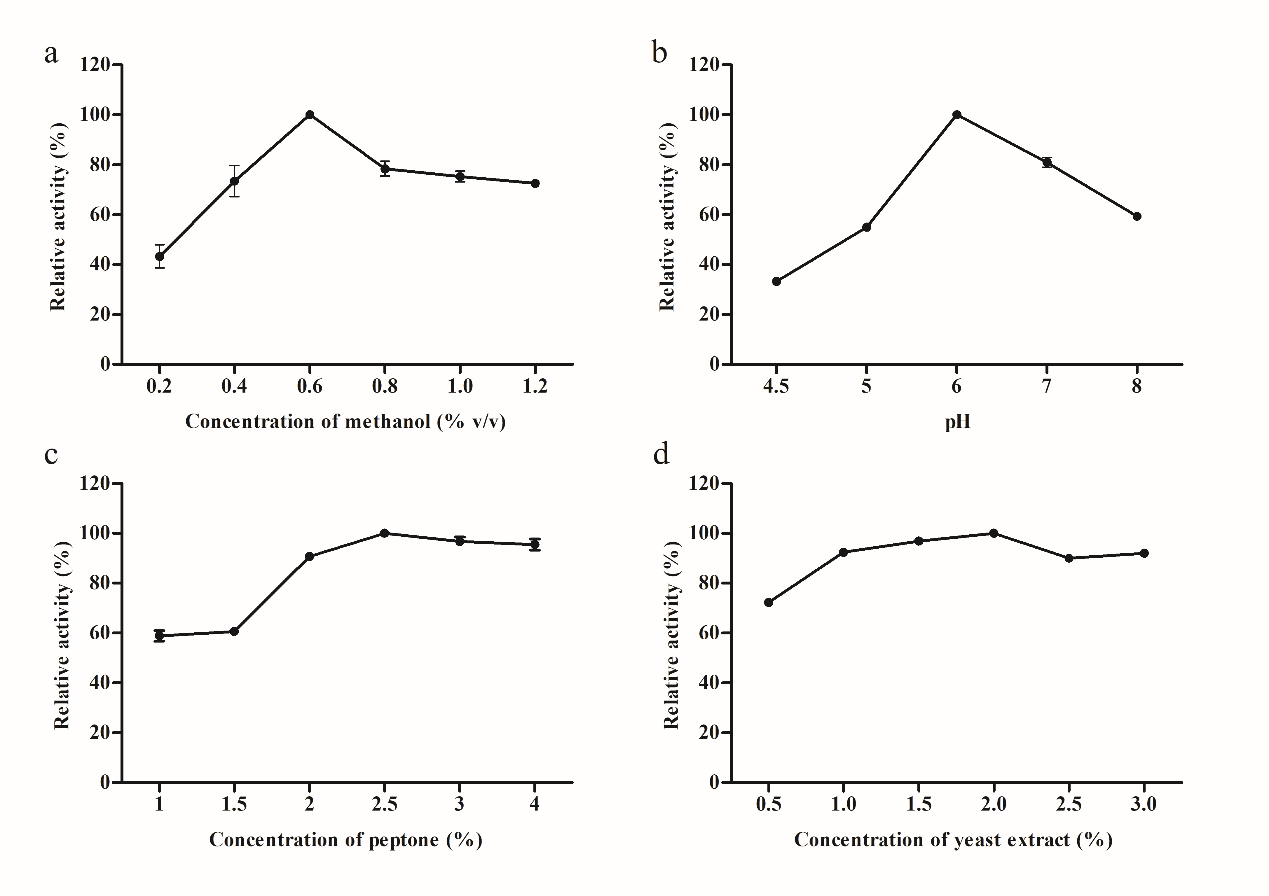


**Fig. S1 the effect of concentration of methanol, pH, concentration of peptone and concentration of yeast extract on enzyme production by recombinant GS115-AmRha strain**


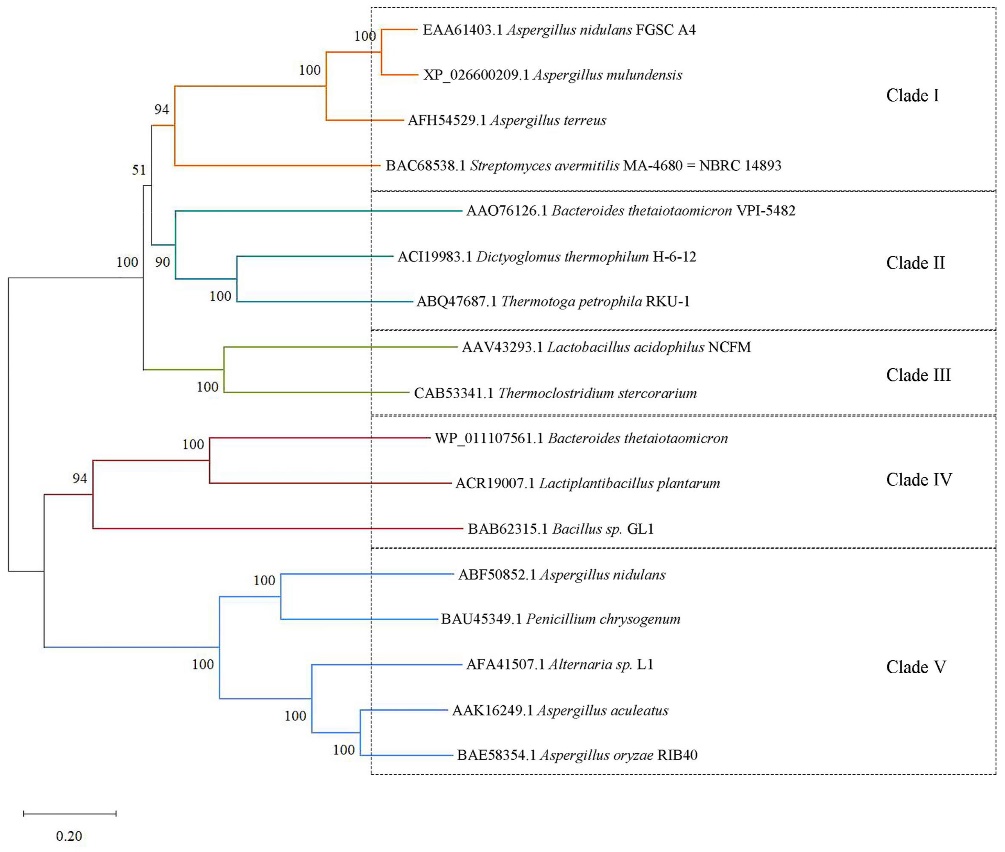


**a). The phylogenetic tree of *α*-L-rhamnosidases from different sources by neighbour-joining method.**

**b). The amino acid sequence alignment of *α*-L-rhamnosidases.**

**Fig. S2 Enzyme screening and bioinformatics analysis of AmRha.**
